# Extraskeletal myxoid chondrosarcoma of the masticator space in a pediatric patient

**DOI:** 10.4317/jced.53888

**Published:** 2017-06-01

**Authors:** Mário-José Romañach, Román Carlos, Michel Nuyens, Bruno-Augusto-Benevenuto de Andrade, Oslei-Paes de Almeida

**Affiliations:** 1 DDS, PhD, Oral Pathology, Department of Oral Diagnosis and Pathology, Federal University of Rio de Janeiro School of Dentistry, Brazil; 2 DDS, Division of Pathology, Centro Clínico de Cabeza y Cuello/ Hospital Herrera Llerandi, Guatemala; 3 MD, Division of Otorhinolaryngology - Head and Neck Surgery, Centro Clínico de Cabeza y Cuello/ Hospital Herrera Llerandi, Guatemala; 4 DDS, PhD, Oral Pathology Section, Department of Oral Diagnosis, Piracicaba Dental School, University of Campinas, Brazil

## Abstract

Extraskeletal myxoid chondrosarcoma (EMC) is a malignant soft-tissue neoplasm rarely described in the head and neck region of children and adolescents. We describe a case of EMC affecting the masticator space and a literature review. A 13-year-old boy who presented a large painless, diffuse mass causing progressive midfacial asymmetry of 6 months duration. Histopathological evaluation revealed a multinodular lesion, containing scattered round vacuolated tumor cells dispersed in an abundant myxoid stroma, separated by fibrous septae. Immunohistochemical analysis revealed positivity for vimentin, neuron-specific enolase, and chromogranin. The Ki-67 labelling index was 42%. The patient was treated surgically with tumor resection followed by adjuvant local radiotherapy. The patient died 1 year after initial diagnosis due to locoregional tumor dissemination. EMC should be considered in the differential diagnosis of myxoid neoplasms in the head and neck region.

** Key words:**Extraskeletal myxoid chondrosarcoma, masticator space, parapharyngeal space, immunohistochemistry, children.

## Introduction

Extraskeletal myxoid chondrosarcoma (EMC) is a rare myxoid tumor accounting for less than 3% of all soft tissue sarcomas (1). Despite the terminology, EMC does not present convincing evidence of cartilaginous differentiation, and recently, a neuroectodermic origin has been proposed (2). Most cases exhibit the specific reciprocal translocation t(9;22) (3).

EMC mainly affects the lower limbs of male patients in their fifth and sixth decades of life, presenting as a painful and tender soft-tissue swelling (1-3). Rarely, EMC occurs in pediatric patients (4) or in other anatomical sites including the head and neck region (3). EMC shows a high signal in T2 weighted magnetic resonance images (MRI), being directly proportional to its myxoid component (5).

Microscopically, the tumor cells of EMC typically present eosinophilic granular, frequently vacuolated cytoplasm with round to oval nuclei, morphologically resembling lipoblasts, immersed in a myxoid stroma in a multilobular arrangement, which is separated by fibrous septae (6-7). The immunophenotype of EMC include positivity for vimentin and variable staining for S-100 and neuroendocrine markers (3,6).

The most effective treatment for EMC is wide surgical excision since most of the cases show poor response to chemotherapy and radiotherapy (7). Local recurrence and distant metastases are common, with some cases presenting a prolonged survival (1,6-7).

To the best of our knowledge 33 EMC cases of the head and neck region have been reported in the literature, most of them affecting the neck, nasal cavity and intracranial cavity ( Table 1) (2,7-33). Only four cases affected pediatric patients, (9,20,25,29) with two previous cases affecting the infratemporal fossa and parapharyngeal space (19,21).

**Table 1:**
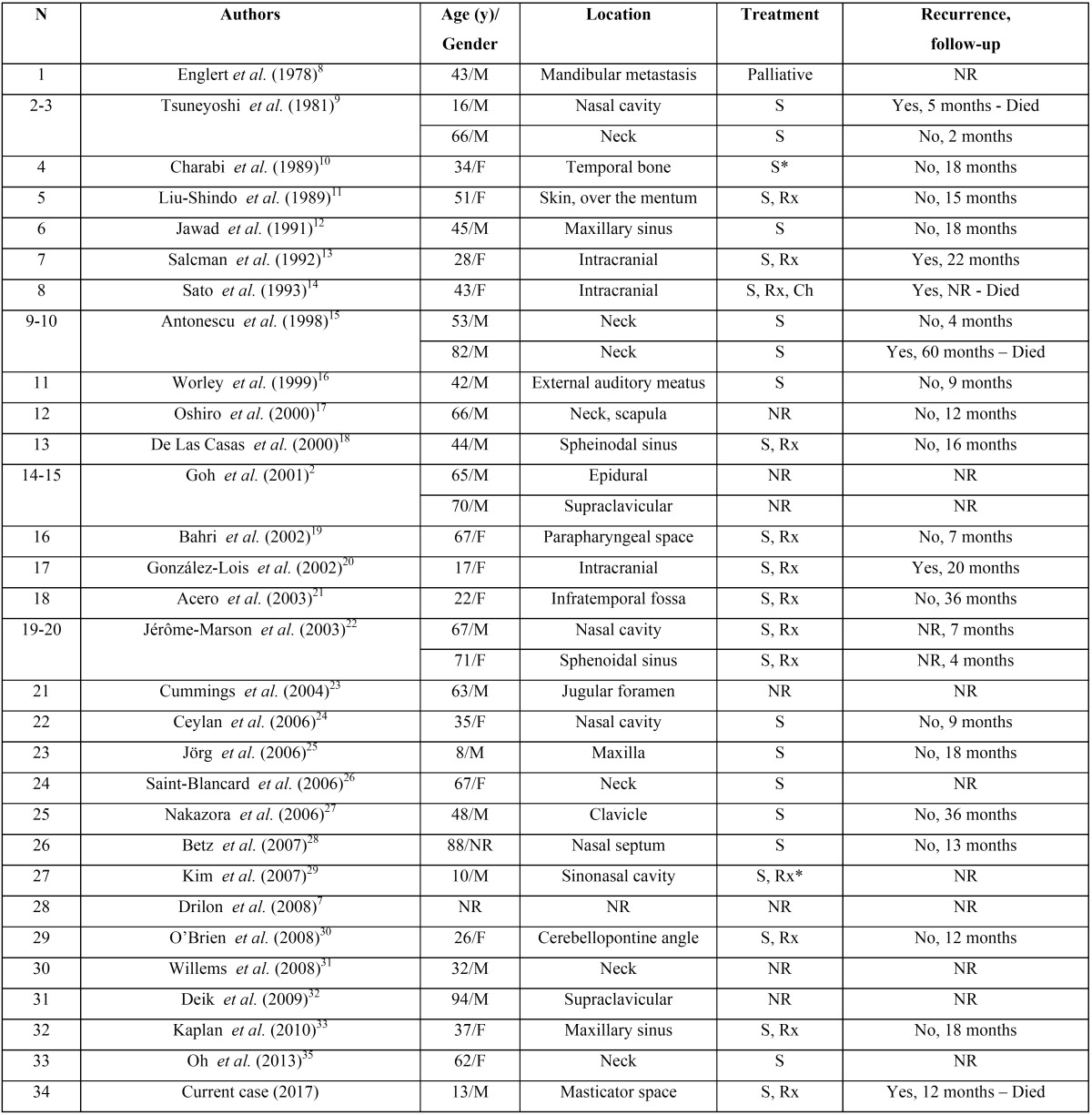
Clinical features of 34 cases of extraskeletal myxoid chondrosarcomas of the head and neck region reported in the English language literature and the present case.

## Case Report

**Figure 1:**
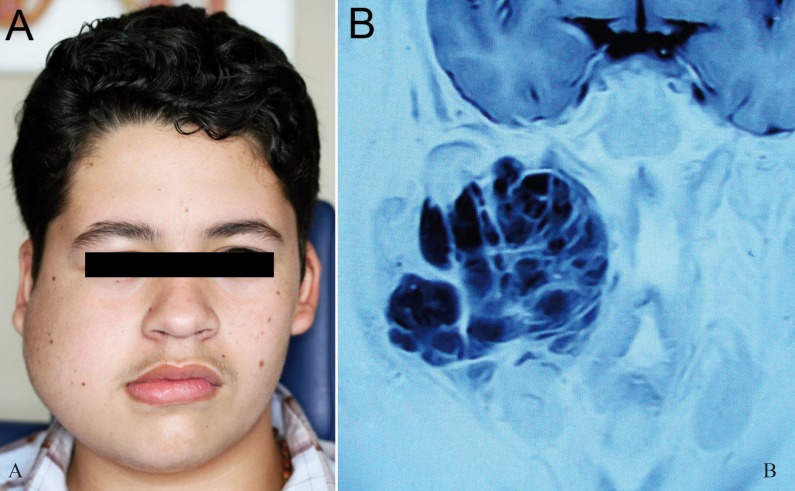
Clinical and imaging features of extraskeletal myxoid chondrosarcoma: (A) Patient with EMC of the masticator space showing a large painless swelling involving the right midface and parotid region. Note the deviation of the right commissure. (B) Inverted image of contrasted T2-wheighted MRI showing a large multinodular tumor with trabecular configuration infiltrating adjacent structures (coronal section).

A 13-year-old male presented with a rapid enlarging painless diffuse mass located at the right midface and parotid region over the last 6 months. Slight asymmetry of the right commissure, trismus and mandibular asymmetry were evident on initial examination. There were no evidences of regional or distant metastasis. Past medical history included a previous surgical treatment attempt in his native country Honduras, three months prior to his referral (Fig. 1A). T1 weighted MRI showed a large hyperintense mass measuring 7 x 5 cm, located centrally at the right masticator space with extention into the mandibular ramus, masseter and pterigoyd muscles, parotid gland and parapharyngeal space. Contrast T2 weighted MRI exhibited a high signal with a multinodular and infiltrative pattern (Fig. 1B).

**Figure 2:**
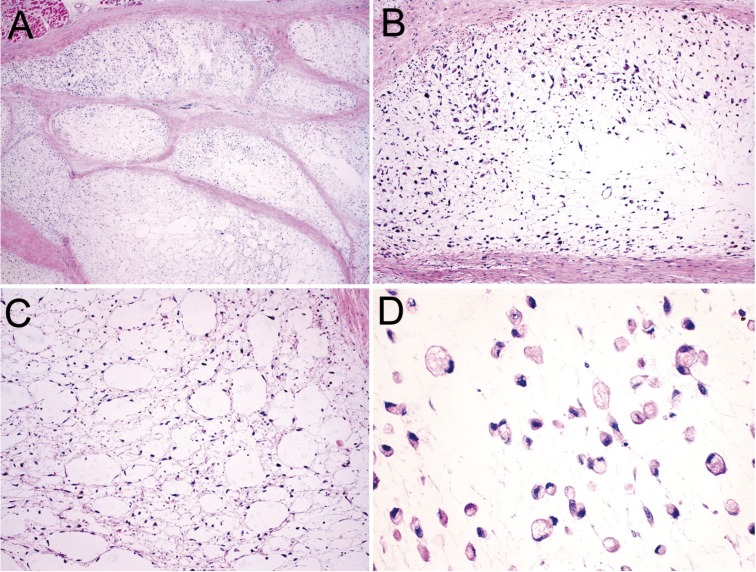
Microscopical features of extraskeletal myxoid chondrosarcoma: (A) Myxoid tumor nodules separated by fibrous septae, containing neoplastic cells (HE, x25). (B) Oval to round tumor cells of different sizes showing lightly eosinophilic to clear cytoplasm within a myxoid stroma. Hypercellular areas are distributed along the periphery of the myxoid nodules (HE, x100). (C) Areas of the tumors contain round to ovoid empty spaces resembling adipose tissue on lower power. (HE, x100). (D) Detail of lipoblast-like cells presenting eosinophilic, vacuolated cytoplasm and round displaced nuclei (HE, x400).

**Figure 3:**
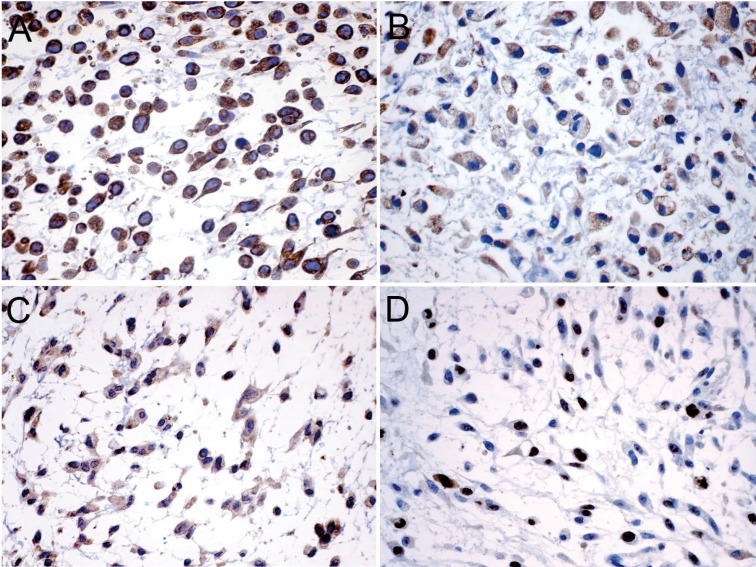
Immunohistochemical profile of extraskeletal myxoid chondrosarcoma: (A) vimentin (IHC, x400), (B) neuron-specific enolase (IHC, x400), (C) chromogranin (IHC, x400). (D) Nuclear staining for Ki-67 with index labeling of 42% (IHC, x400).

An incisional intraoral biopsy was performed under local anesthesia. The histopathological examination revealed multiple nodules containing tumor cells, immersed in an abundant myxoid stroma separated by fibrous septae. The tumor cells varied in shape from round to elongate, most displaying abundant eosinophilic cytoplasm and round to ovoid excentrically located nuclei, which showed homogeneously distributed chromatin and inconspicuous nucleoli. Other cell types were recognized, mainly spindle, epithelioid and lipoblast-like cells (Fig. 2). The mitotic rate was low, averaging two mitotic figures per 10 high-power fields. Periodic acid-Schiff (PAS), with and without prior diastase digestion, demonstrated that the granular cytoplasmic material was glycogen. Immunohistochemical analysis ( Table 2), showed positivity for vimentin, neuron-specific enolase (NSE), and chromogranin; whereas AE1/AE3, epithelial membrane antigen (EMA), S-100, desmin, muscle-specific actin (HHF35), CD57, Glucose transporter type 1 (Glut-1), synaptophysin, p53, p63, glial fibrillary acidic protein (GFAP), and podoplanin (D2-40) were negative. The Ki-67 labelling index was 42% after evaluation of 1000 cells per five high-power fields (Fig. 3). Fluorescent in situ hybridization was not performed; however, the histomorphological and immunohistochemical findings supported a final diagnosis of EMC.

**Table 2:**
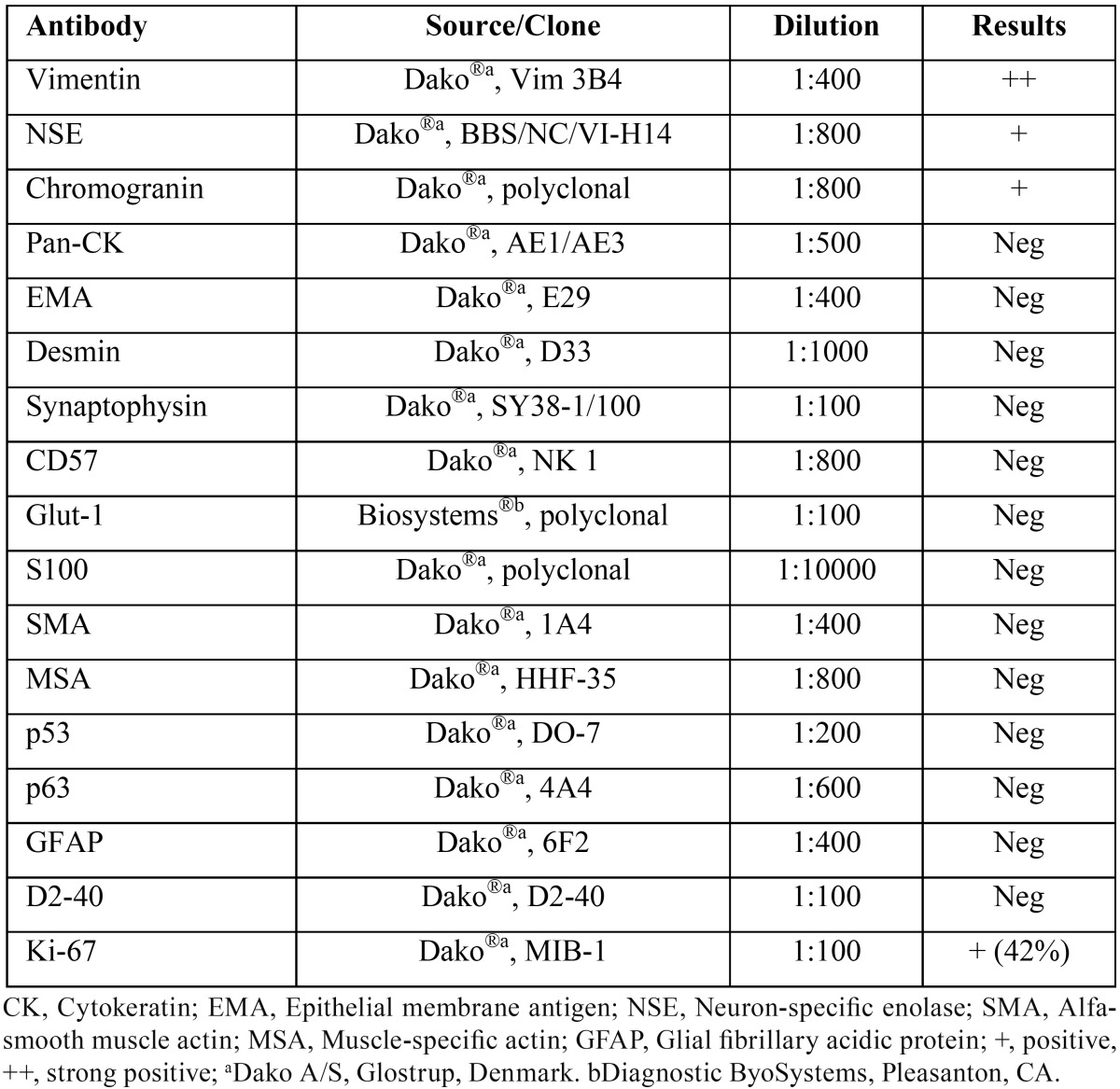
Antibodies used for immunohistochemistry and results positive/negative in extraskeletal myxoid chondrosarcoma of the masticator space.

There were no evidences of regional or distant metastases, and the patient was treated with en-bloc surgical resection, including ipsilateral hemi-mandibulectomy, total parotidectomy with facial nerve dissection, neck dissection level I to IV and reconstruction with a mandibular plate and major pectoralis flap. Macroscopically, the specimen measured 8 x 6 x 5 cm consisting of an irregularly shaped mass attached to the mandibular ramus, with a multilobular and gelatinous rubbery tissue. Focally, the tumor was present in one surgical margin, immediately anterior to the mandibular condyle. Microscopic evaluation revealed the same histopathological features found in the incisional biopsy. The patient received adjuvant radiotherapy but died after 1 year of follow-up due to complications of locoregional tumor dissemination to the skull base.

## Discussion

Extraskeletal myxoid chondrosarcoma (EMC) is a rare myxoid soft tissue sarcoma first described as a distinct entity by Enzinger & Shiraki (1972) (2-3). Since the initial description, skeletal myxoid chondrosarcoma and EMC are considered different entities; (34) in fact, the latter does not show convincing evidences of cartilaginous origin (1-3). Moreover, most EMC present the reciprocal translocation t(9;22)(q22;q12) that recombines the genes nuclear receptor subfamily 4, group A, member 3 (NR4A3, also termed TEC, NOR1 or CHN [9q22]) and Ewing sarcoma region 1 (EWS [22q12]).2,8 Other cytogenetic subgroups has been less commonly described, including t(9;17)(q22;q11) and t(9;15)(q22;q21) (2,3,8).

EMC of the head and neck region is rare, with only 33 cases reported in the English language literature to this date, including the present case ( Table 1). In the head and neck region, the most common sites of EMC are the nasal cavity and neck, followed by intracranial cavity, clavicular region, maxilla, sphenoid sinus and temporal bone. To the best of our knowledge, there is only one previous report of EMC involving the masticator space (21). Most of the patients presenting EMC are in their fifth to seventh decade of life. At present, there are only 3 reported cases affecting the oral and maxillofacial region in pediatric patients, (25,29) including our case. Tumors involving the facial region are usually diagnosed after reaching large size, destroying underlying bone with invasion into the subcutaneous soft tissues (20-21,29). The most commonly accepted treatment is surgical resection, but due to technical complexities related to this anatomical region, complete tumor resection often is not achieved, resulting in poorer prognosis(20-21). In the present case, the superior margin was microscopically compromised, and the adjuvant radiation therapy did not prevent local tumor dissemination, causing the death of the patient. Nevertheless, Acero *et al.* (21) reported a case of EMC affecting the infratemporal fossa and masticator space of a 22-year-old female patient, which was treated by surgical excision followed by adjuvant radiotherapy and no signs of recurrence was seen 3 years of post-treatment follow-up.

Imaging features of EMC include a multi-nodular soft tissue proliferation with high intensity signal on T2-weighted MRI and heterogeneous enhancement on contrasted images, (5) as observed in the present case. Grossly, the majority of EMC presents as a large well demarcated tumor with nodular areas of gelatinous appearance, (5,9) as also found in the present case.

Microscopically the nodular gelatinous areas correspond to the myxoid stroma, demarcated by fibrous septa (1,3). Tumor cells are immersed on this matrix, isolated or more typically forming interconnecting cords or clusters. The cells show granular or vacuolated eosinophilic cytoplasm, containing round nuclei with inconspicuous nucleoli (1-3,6). Characteristically, diastase-sensitive PAS positive staining demonstrates glycogen in the tumor cells. The classic EMC morphology might not be seen in some cases, where more cellular areas with minimal myxoid matrix and tumor cells with epithelioid, rhabdoid and spindle cell features are observed (3). Mitotic figures are uncommon while intratumoral haemorrhage can be observed (1,3,6). The present case showed the classic morphology of EMC, which facilitated the diagnosis.

The EMC immunophenotype includes diffuse positivity for vimentin (77%), focal positivity for synaptophysin (59%), NSE (54%) and S-100 protein (31%), while chromogranin is expressed in a minority of cases. Epithelial markers such as cytokeratins and EMA are usually negative (2,6). Expression of these markers and/or demonstration of neuro-secretory granules by electron microscopy support the hypothesis of a possible neuroendocrine differentiation (3), indicating that the term chondrosarcoma is inappropriate. The present case showed the typical histological morphology, and positivity for vimentin, NSE and chromogranin, confirming the diagnosis. The Ki-67 index found on this case can be considered high (42%), explaining in part the aggressive clinical behavior, however, the affected anatomical region and initial incomplete surgical treatment also contributed to the fatal disease progression. The extraskeletal site was supported by imaging evaluation, and was confirmed during the surgical procedu-re.

EMC may show typical, alternating hypercellular and hypocellular areas, and these aspects should be considered in the differential diagnosis (6). As for cases of typical EMC, our histological differential diagnosis included myxoid tumors such as chordoma, parachordoma, myxoid liposarcoma, and myxofibrosarcoma. Because of the patient´s age and the epithelioid morphology of some tumor cells, embryonal rhabdomyosarcoma, epitheliod sarcoma and extrarenal rhabdoid tumor were also considered, but as less likely possibilities. Histological characteristics as absence of physalliferous cells, elongated curvilinear blood vessels, salivary gland duct-like structures, rhabdomyoblasts, globoid cytoplasmic inclusions of rhabdoid cells and cytokeratin, S-100 and myogenic markers negativity helped to exclud these possibilities (48). In EMC cases with non-classic morphology or hypercellular-predominant areas, molecular studies such as FISH or RT-PCR may be required to confirm the diagnosis (2,3).

As mentioned above, the treatment of choice for patients with EMC is surgical tumor resection with clear margins (7,21). Alt-hough EMC was previously considered a low-grade sarcoma, currently its prognosis has been reported as unfavorable with a high mortality rate, particularly for pediatric patients and tumors larger than 10 cm (1,3-4). Almost half of EMC cases present local recurrence and distant metastasis, despite the estimated 5 years survival rate of 90% (6). In cases with microscopically positive margins, high doses of adjuvant radiation therapy have been useful for the control of local tumor dissemination in contrast with chemotherapy, which plays limited or no role (7). In the present case, the surgical resection of a large tumor involving the masticator space of a pediatric patient was technically difficult, and was followed by adjuvant radiotherapy. Unfortunately, the patient died after 1 year of follow-up due local recurrence of disease.

In summary, we report a rare case of EMC affecting the masticator space and adjacent structures of a pediatric patient with unfavorable outcome. Clinicians and pathologists should be aware to consider EMC in the differential diagnosis of myxoid neoplasms in the head and neck region.
